# Univariate Statistical Analysis as a Guide to ^1^H-NMR Spectra Signal Assignment by Visual Inspection

**DOI:** 10.3390/metabo9010015

**Published:** 2019-01-15

**Authors:** Chenglin Zhu, Beatrice Vitali, Gilbert Donders, Carola Parolin, Yan Li, Luca Laghi

**Affiliations:** 1 Department of Agri-Food Science and Technology, University of Bologna, 40126 Bologna, Italy; chenglin.zhu2@unibo.it (C.Z.); yan.li7@unibo.it (Y.L.); 2 Department of Pharmacy and Biotechnology, University of Bologna, 40126 Bologna, Italy; b.vitali@unibo.it (B.V.); carola.parolin@unibo.it (C.P.); 3 Department of Obstetrics and Gynaecology, General Hospital Heilig Hart, 3000 Tienen, Belgium; gilbert.donders@femicare.net; 4 Department of Obstetrics and Gynecology, Antwerp University, 2000 Antwerp, Belgium

**Keywords:** ^1^H-NMR, metabolomics, signals assignment, visual inspection

## Abstract

In Proton Nuclear Magnetic Resonance (^1^H-NMR) spectroscopy, the signals assignment procedure is normally conducted by visual inspection of the spectra, by taking advantage of the innate predisposition of human eye for pattern recognition. In the case of untargeted metabolomics investigations on food and body fluids, the complexity of the spectra may lead the user to overlook signals, independently from their biological relevance. Here, we describe a four steps procedure that is designed to guide signals assignment task by visual inspection. The procedure can be employed whenever an experimental plan allows for the application of a univariate statistical analysis on a point-by-point basis, which is commonly the case. By comparing, as a proof of concept, ^1^H-NMR spectra of vaginal fluids of healthy and bacterial vaginosis (BV) affected women, we show that the procedure is also readily usable by non-experts in three particularly challenging cases: overlapping multiplets, poorly aligned signals, and signals with very poor signal-to-noise ratio. The paper is accompanied by the necessary codes and examples written in R computational language to allow the interested user gaining a hands-on impression of the procedure’s strengths and weaknesses.

## 1. Introduction

^1^H-NMR spectroscopy represents one of the election techniques for investigating the metabolome of food and body fluids, defined as the ensemble of their small metabolites that were observed comprehensively [1]. In sporadic cases, researchers try to make their observations on the metabolome really comprehensive by treating ^1^H-NMR spectra similarly to fingerprints of the samples under investigation [2,3,4]. They do so by considering the points constituting each spectrum as variables by means of multivariate analysis, with no attempt to ascribe the NMR signals to specific molecules. A far more common alternative to have comprehensive observation of the metabolome is represented by the assignment of as many signals as possible to specific molecules [5].

The task of signals assignment has been faced by the first works dealing with metabolomics by means of two-dimensional (2D) experiments [6]. Registering and interpreting 2D spectra is a labor-intensive task. In addition, the limit-of-detection of 2D-NMR may be 100 times lower than its one-dimensional (1D) counterpart. As a step forward, the creation of public databases, listing chemical shift and multiplicity of the signals of pure compounds, has allowed the high throughput identification of molecules that are based on 1D spectra only. The use of these databases has several limitations, one of them being the need to jump back and forth the software for spectra visualization and the databases. The software product Amix (Bruker, Milan, Italy), launched in 1996, has filled this gap for the first time, by allowing to query the databases within the visualized spectra. Chenomx (Chenomx inc., Edmonton, Canada), represents an evolution of Amix, because it enables the user to interact simultaneously with the spectrum under investigation and with the actual spectra of pure molecules, with that are dynamics typical of the videogames. In this way, Chenomx exploits the innate predisposition of human beings for pattern recognition, similarly to the software/game Foldit (www.fold.it) [7] in the field of protein folding.

**Figure 1:**
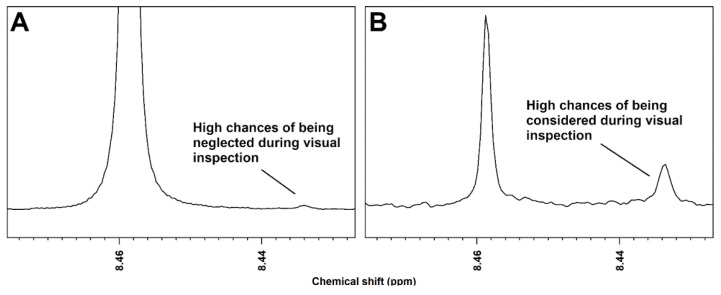
(**A**) A signal with an unfavorable signal-to-noise ratio and surrounded by much higher signals will be probably neglected during visual inspection. (**B**) When signals in spectrum region are of similar intensity, well above the limit of detection, visual inspection is likely to lead to the comprehensive observation of the metabolome.

It must be noticed that the very first step of all the above procedures for comprehensive assignment is represented by the moment when the user focuses the attention on a certain signal and decides that it is worth assigning it. When this action is started by unguided visual inspection, the aforementioned innate predisposition of human beings for pattern recognition may make the assignment not comprehensive at all. In fact, signals with high intensity may attract attention at the expense of the nearby signals, while signals with intensity that is close to the limit of detection may be neglected right away. In addition, the software products for visual inspection of ^1^H-NMR spectra are generally designed to make the user load one spectrum at a time. This exacerbates the two aforementioned problems, because it is unlikely (impossible, in our hand-on experience) that such spectrum has the best signal-to-noise ratio for all the molecules that can be retrieved along the entire set of spectra. Figure 1 gives a visual impression of the three problems.

We have observed that if a set of spectra is suitable for point-by-point univariate analysis, such as *t*-test or ANOVA, the resulting *p*-values can lead to a spectrum-like representation of surprising effectiveness in guiding the operator visual inspection. Here, we outline a ^1^H-NMR signals recognition procedure based on this concept and we employ an exclusively visual approach to point out its strengths and weaknesses. As a case study, we focus on a set of ^1^H-NMR spectra that were acquired on vaginal fluids of healthy and BV affected women, as part of our efforts to better characterize this disease [8,9,10,11,12]. The present work is a step in our ongoing quest for ^1^H-NMR untargeted signals assignment instruments balancing simplicity and performance.

## 2. Results

**Figure 2:**
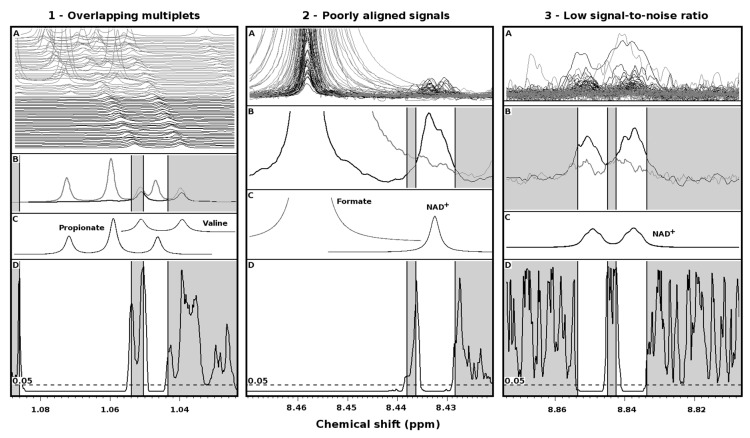
Performance of procedure for the reconstruction of signals by means of point-by-point univariate analysis, represented in the three particularly challenging cases of overlapping multiplets (**1**), poorly aligned signals (**2**) and low signal-to-noise ratio (**3**). Superimposition (**A**) and average (**B**) of portions of the spectra acquired on healthy (black line) and BV-affected (light gray line) women. (**C**) Simulation of the main signals appearing in such portions. (**D**) *P*-values of the point-by-point Wilcoxon tests. White portions identify the reconstructed signals.

The performance of the procedure is illustrated in three extreme cases (Figure 2).

Overlapping multiplets (Figure 2(1)). BV is known to lead to high concentrations of propionate in vaginal fluid [8]. One of the peaks of its triplet at 1.06 ppm appears between valine doublet, centered at 1.04 ppm (panel 1A). Wilcoxon tests performed over each point of the spectra corresponding to propionate (panel 1D) give *p*-values lower than 0.05, so that each peak of its triplet is evidenced by the procedure. At the opposite, in correspondence of valine peaks, no point shows *p*-values lower than 0.05, so that such peaks get automatically discarded.

Poorly aligned signals (Figure 2(2)). The procedure based on Wilcoxon *p*-value correctly identifies the signal at 8.45 ppm of formate (panel 2D), because more concentrated in vaginal fluid of BV affected women. More interestingly, the procedure identifies one of the signals from NAD^+^, even if poorly aligned and in some cases even overlapped with one tail of the main peak of formate.

Poor signal-to-noise ratio (Figure 2(3)). The NAD^+^ signal between 8.82 and 8.86 (panel 3A) would pass unnoticed at an unguided visual inspection of most of the spectra, observed one by one. The same signal would have more chances of grabbing attention, if the plot of the average spectra was observed (panel 3B). The procedure based on the *p*-value nicely identifies the signal as a doublet (panel 3D), thus confirming the assignment of NAD^+^ signal shown in the previous paragraph.

## 3. Discussion

Several scientific journals publish works that describe algorithms and procedures for exploring ^1^H-NMR spectra from the point of view of metabolomics. These algorithms have been of great value for shedding light on a wealth key topics, such as oncology [13] and nutrition [14], by focusing on several biofluids, such as feces [15] or urine [16]. A part of them approaches the identification of molecules untargetedly. A query to scholar (https://scholar.google.it) with the keywords “NMR”, “metabolomics”, and “untargeted” returns for 2018 more than 2K works. Among them, we could find none where the procedure for untargeted identification of the molecules is described in sufficient detail. This suggests that the authors of those papers have conducted such identification in an unguided fashion, exploring back and forth the spectra to spot as many molecules as possible. This also suggests that the potential reduction of molecules assigned caused by the visual inspection step is a problem that is still untouched, so that the present work could be considered to be a primer in this respect.

The everyday work with software products devoted to ^1^H-NMR spectra interpretation suggests that the key point of any effective method for signals assignment is simplicity of use. The present work describes a procedure to guide signals assignment where the most difficult step is plotting *p*-values together with spectra. The *p*-values may come from any univariate statistical analysis when comparing two groups (i.e., *t*-test, Wilcoxon-test) or more than two groups (i.e., ANOVA, Kruskal Wallis). Despite the extreme simplicity, Figure 2 demonstrates that the procedure could make the difference in particularly complicated situations where signals alignment, signals superimpositions, or signal-to-noise are far away from ideal. About the latter problem, Table 1 of the work by Vitali et al. [9] shows that, among the 32 molecules that have been found to differ between healthy and BV-affected women, as many as six (20%) have a signal-to-noise ratio similar to the one of NAD^+^.

## 4. Materials and Methods

### 4.1. Samples, Spectra and Statistics

Based on criteria that are described in greater detail elsewhere [12], 43 women affected by bacterial vaginosis (BV) and 37 age-matched healthy women (H) were recruited. Samples were prepared, as described by Laghi and Vitali [8,9]. In detail, ^1^H-NMR spectra were recorded at 298 K with an AVANCE spectrometer (Bruker, Milan, Italy) operating at a frequency of 600.13 MHz. The HOD residual signal was suppressed by applying the first increment of the NOESY pulse sequence and a spoil gradient. Each spectrum was acquired using 32 K data points over a 7211.54 Hz spectral width and adding 256 transients. A recycle delay of 5 s and a 90° pulse of 11.4 μs were set up.

All of the calculations were performed in R language (www.R-project.org). Following the choice of Vitali et al. [9], statistically significant differences between the spectra from BV and H groups were looked for by the Wilcoxon test, performed on a point-by-point basis. Simulations of spectra from pure compounds were performed in Chenomx (ver 8.0).

### 4.2. Rationale of the Procedure for Signals Reconstruction

**Figure 3:**
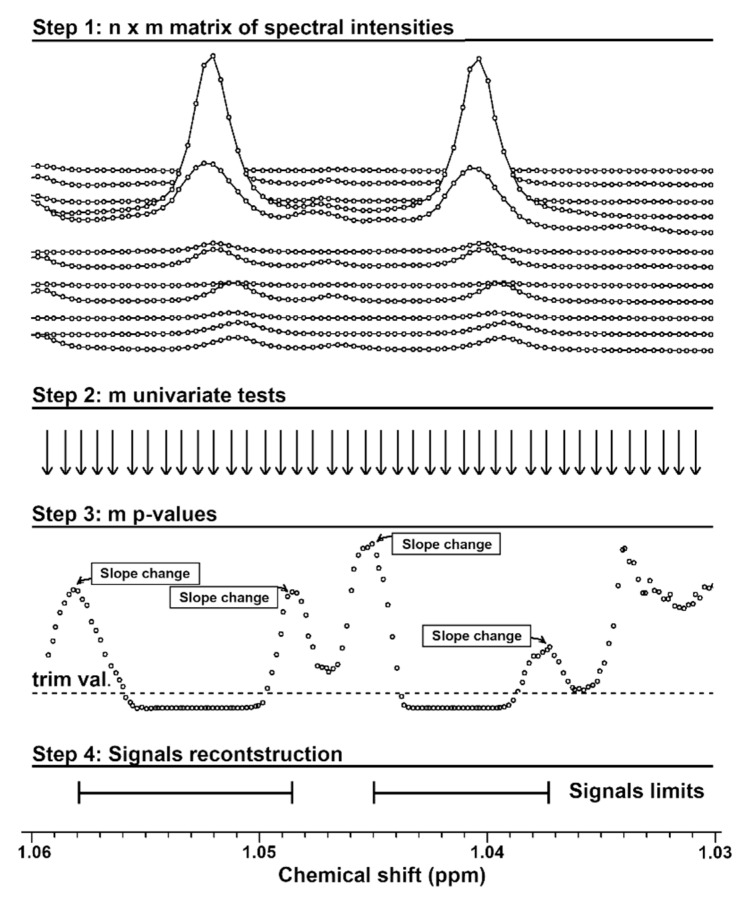
Representation of the procedure for the reconstruction of signals by means of point-by-point univariate analysis.

The rationale of the procedure for the reconstruction of signals by means of point-by-point univariate analysis is represented graphically in Figure 3. Given that the intensities of n NMR spectra of m points are stored in a n × m matrix (step 1), a univariate statistical analysis (Wilcoxon test, in the present case) can be applied on each column of the matrix (step 2), to create a vector of *p*-values of length m. If the *p*-values are drawn below the stack plot of the spectra (step 3), then it can be noticed that they lead to a pseudo-NMR spectrum, with peaks pointing downward. The reason is that the *p*-values tend to reach a minimum at the center of every signal of a molecule that is highly significant in the statistical analysis applied, while the adjacent points show, progressively, higher *p*-values. Consequently, the extremes of the signal fall empirically in correspondence of the first sign change of the slope of the *p*-values curve (step 4).

### 4.3. A Hands-on Example

The user interested in going through each of the four steps of the procedure with a live example can be found in the supplementary material, where the necessary resources are written in R-language. (Steps 1–3) The text file “Unifind.example.txt” contains the experimental points of one spectrum per group of women and the *p*-values from the Wilcoxon test originally performed for the work by Vitali et al. [9]. (Step 4) Signals reconstruction, spectra visualization, and *p*-values analysis are possible through the R script “unifind.R”, accompanied by a detailed manual. The example provided in the manual will guide the reader through each step.

## 5. Conclusions

Metabolomics has born to give qualitative and quantitative information about all the low weight molecules that are present in a biofluid, representing the so called metabolome space [17]. Obviously, any platform is severely limited in the possibility to explore completely such space due technical characteristics. It is less obvious that there can be space for improvements that does not involve costly technical upgrades. One of them is represented by not ignoring information actually available. This work makes a procedure guiding visual inspection of ^1^H-NMR spectra available, a key step of molecules characterization that is often taken too much for granted.

## Supplementary Materials

Supplementary materials can be found at http://www.mdpi.com/2218-1989/9/1/15/s1.
