# Supplementary material for: Univariate Statistical Analysis as a Guide to 1H-NMR Spectra Signal Assignment by Visual Inspection
Source: Metabolites. 2019 Jan 15;9(1):15. doi: 10.3390/metabo9010015 (PMC6359365; doi:10.3390/metabo9010015)
Supplement: Supplementary file 1 [file metabolites-09-00015-s001.zip › Unifind_manual.pdf]

Highlight NMR spectra signals according to univariate analysis

Description

Unifind reconstructs the NMR spectra signals by taking advantage of the p-value of univariate analysis applied on a point-b-point basis.

Usage

```
unifind(x, y, P1, draw.spectra=T, ...)
```

Arguments

`x`	vector with the spectra chemical shift. Missing values (NAs) are allowed.
`y`	matrix of the spectra intensities. The number of columns must match x length. Missing values (NAs) are allowed.
`P1`	vector of p-values from univariate analysis. Missing values (NAs) are allowed.
`trim`	Vector of length 1 with the desired p-value to be used as a trim.
`draw.spectra`	logical. If TRUE, spectra are plotted and signals reconstructed by unifind are evidenced with gray shadows in black boxes.
`draw.p`	logical. If TRUE, p-values are superimposed to the spectra and the trim value is highlighted.
`...`	Graphical parameters (see matplot) passed to matplot R function.

Value

`signals.points`	logical vector characterized by TRUE values in correspondence with signals reconstructed by unifind. Its length is equal to that of x.
`signals.extremes`	two columns matrix with the extremes of the signals reconstructed by unifind.

Details

The function handles y matrix also when entire columns are constituted by NA's. The same applies to P1 vector. This is the case with spectra matrices where the signals have been aligned.

See Also

`matplot`

Examples

```
unifind.example<-as.matrix(read.table("unifind.example.txt"))
probability.vector<-unifind.example[1,]
ppm.vector<-unifind.example[2,]
spectra.matrix<-unifind.example[3:4,]
source("unifind.R")
Out.object<-unifind(ppm.vector, spectra.matrix, probability.vector,
draw.spectra=T, xlim=c(1.09,1), ylim=c(0,0.4), draw.p=T)
axis(3, at=c(1.072633, 1.059625, 1.046969, 1.019547, 1.007594), labels=F)
axis(3, at=c(1.0596, 1.012867), labels=c("Propionate","Isoleucine"),
tick=F)
legend(1.092145, 0.405269, legend=c("spectrum 1", "spectrum 2", "p-
values"), col=c(1,2,3), pch=16, bg="white")
```
